# Hippocampal capillary pericytes in post-stroke and vascular dementias and Alzheimer’s disease and experimental chronic cerebral hypoperfusion

**DOI:** 10.1186/s40478-024-01737-8

**Published:** 2024-02-15

**Authors:** Yoshiki Hase, Dan Jobson, Jeremy Cheong, Kelvin Gotama, Luciana Maffei, Mai Hase, Alhafidz Hamdan, Ren Ding, Tuomo Polivkoski, Karen Horsburgh, Raj N. Kalaria

**Affiliations:** 1 Neurovascular Research Group, Translational and Clinical Research Institute, Campus for Ageing & Vitality, Newcastle University, NE4 5PL Newcastle upon Tyne, UK; 2 Centre for Neuroregeneration, University of Edinburgh, Little France Crescent, Edinburgh, UK

**Keywords:** Alzheimer’s disease, Brain capillary, Collagen IV or COL4, Dementia, Hippocampus, Pericyte, Vascular dementia

## Abstract

Neurovascular unit mural cells called ‘pericytes’ maintain the blood-brain barrier and local cerebral blood flow. Pathological changes in the hippocampus predispose to cognitive impairment and dementia. The role of hippocampal pericytes in dementia is largely unknown. We investigated hippocampal pericytes in 90 post-mortem brains from post-stroke dementia (PSD), vascular dementia (VaD), Alzheimer’s disease (AD), and AD-VaD (Mixed) subjects, and post-stroke non-demented survivors as well as similar age controls. We used collagen IV immunohistochemistry to determine pericyte densities and a mouse model of VaD to validate the effects of chronic cerebral hypoperfusion. Despite increased trends in hippocampal microvascular densities across all dementias, mean pericyte densities were reduced by ~25–40% in PSD, VaD and AD subjects compared to those in controls, which calculated to 14.1 ± 0.7 per mm capillary length, specifically in the cornu ammonis (CA) 1 region (*P* = 0.01). In mice with chronic bilateral carotid artery occlusion, hippocampal pericyte loss was ~60% relative to controls (*P <* 0.001). Pericyte densities were correlated with CA1 volumes (r = 0.54, *P* = 0.006) but not in any other sub-region. However, mice subjected to the full-time environmental enrichment (EE) paradigm showed remarkable attenuation of hippocampal CA1 pericyte loss in tandem with CA1 atrophy. Our results suggest loss of hippocampal microvascular pericytes across common dementias is explained by a vascular aetiology, whilst the EE paradigm offers significant protection.

**Supplementary Information:**

The online version contains supplementary material available at 10.1186/s40478-024-01737-8.

## Introduction

Brain vascular injury is a major risk factor for dementia. Longitudinal follow up studies in cognitively intact elderly stroke survivors suggests up to 50% will go on to develop delayed post-stroke dementia (PSD) [40, 41]. Using structural and arterial spin labelling magnetic resonance imaging (MRI) methods, we previously found that medial temporal lobe atrophy is an important predictor of PSD [13, 15]. Hippocampal volumes were also reduced almost equally in PSD as in patients diagnosed with Alzheimer’s disease (AD) relative to both post stroke no-dementia (PSND) and ageing control subjects [14]. Similarly, moderate to severe medial temporal lobe atrophy was present in more than 40% of cognitively impaired patients with cerebral small vessel disease [2, 27].

Our previous pathological studies showed that volumes of hippocampal neurons in the cornu ammonis (CA) 1, CA2 and CA4 regions were reduced in PSD, vascular dementia (VaD) and AD as well as mixed AD plus VaD subjects relative to PSND and ageing controls [17, 18]. The hippocampal changes and atrophy are explained as occurring remotely from the primary stroke probably due to diaschisis. The focal loss and shrinkage of hippocampal subfield neurons was also correlated with memory scores in the general absence of substantial burdens of neurodegenerative pathology. These observations suggest there is a vascular basis for hippocampal neurodegeneration which concurs with the neuroimaging findings on hippocampal atrophy even in population-based incident VaD [44]. The atrophy may occur due to chronic hypoperfusion affecting the hippocampal vasculature [25] producing neuronal or dendritic arbour loss with consequences to connectivity and function. Moreover, changes in hippocampal haemodynamics may be critical in altering cellular components [25]. However, it is unclear how other key cellular components of the neurovascular unit such as pericytes are modulated in cognitively stable stroke survivors or those who develop PSD and VaD. Capillary pericytes are particularly susceptible to ischaemic injury and associated with hippocampal blood brain barrier breakdown [20, 34]. Previous studies have used platelet-derived growth factor (PDGF)-receptor β immunoreactivity to track pericyte coverage in various conditions [33] but the absence of more reliable markers has hampered greater characterisation of pericytes.

We used a simple reliable method using collagen IV (COL4) immunohistochemistry to identify nucleated pericytes and determined their status in PSD, VaD, AD and mixed dementia with vascular and Alzheimer pathologies in relation to capillary density and hippocampal atrophy. This study elucidated capillary pericytes within specific hippocampal sub-regions and tested whether chronic hypoperfusion produced by bilateral carotid artery stenosis (BCAS) in mice produces similar hippocampal effects that can be modified by environmental enrichment (EE). Our focus particularly on the hippocampus and its vasculature provides an intervention target to mitigate common dementias.

## Materials and methods

### Human subjects

Table 1 provides the demographic details and diagnoses of the total number of subjects used in this study. The PSND, PSD and VaD groups were derived from the Newcastle Cognitive Function After Stroke (CogFAST) study [1]. Dementia was clinically diagnosed and pathologically verified by post-mortem examination as either PSD, AD, VaD or AD-VaD (Mixed). Available radiological reports indicated typical features of dementia [23]. In addition, we assessed PSND subjects as well as ageing controls. The controls, aged 72–91 years, were obtained either from prior prospective studies or other brain donations to the Newcastle Brain Tissue Resource (NBTR). Apolipoprotein E (*APOE*) allele frequencies were determined in frozen samples from the NBTR essentially as described previously [3]. Ageing controls had no evidence of cognitive impairment, clinical or pathological features of neurological or psychiatric disease. Local research ethics committees at the Newcastle upon Tyne NHS Foundation Hospitals Trust granted the necessary ethical approvals for this post-mortem research. Permission for using brains was also granted by informed consent from the individuals themselves when they had been still alive or from a next-of-kin family member. All brain tissues were obtained from the NBTR.

**Table 1:** Demographic details of all the cases and controls

Variable		Ageing Controls	PSND	PSD	VaD	Mixed	AD
N		13	22	13	16	13	13
Mean Age, years (range)		80.1 (72–91)	83.5 (78–94)	87.3 (80–98)	86.9 (76–97)	85.9 (72–94)	83.3 (70–91)
Gender (M:F%)		35:65	57:43	30:70	41:59	44:56	56:44
MMSE, mean ± SEM		29 ± 1	27 ± 0.4	16 ± 1	13 ± 4	11 ± 2	7 ± 2
CAMCOG, mean ± SEM		na	90 ± 1	66 ± 3	na	na	39 ± 7
*APOE ε2; ε4* allele frequencies (%)		16.7; 16.7	10.0; 25.0	13.3; 13.3	10.0; 0.0	0.0; 30.0	3.6; 39.3
CERAD, mean (range)		0.5 (0–2)	1.7 (1–2)	1.3 (1–3)	1.0 (0–2)	2.9 (2–3) ‡	2.9 (2–3) ‡
ABC Scores, mean		A0.5, B1.2, C0.5	A0.5, B1.2, C0.7	A0.5, B1.2, C0.8	A0.6, B1.2, C0.8	A2.5, B2.6, C2.6	A3, B3, C3
Braak Stage, mean (range)		1.9 (0–4)	2.6 (1–4)	2.6 (1–4)	2.0 (0–4)	5.2 (5–6) ‡	5.6 (5–6) ‡
CAA- frequency (moderate- severe), %		6	15	18	17	9	39
Vascular pathology score, mean (range)†		6.7 (0–10) †	13.5 (13–14)	13.3 (9–17)	13.2 (10–16)	11.0 (6–14)	10.8 (3–16)
WML score, mean (range)		0.5 (0–2) †	2.5 (2–3)	2.4 (2–3)	2.9 (2–3)	2.9 (2–3)	1.8 (0–3)
White matter (WM) / Vascular lesions, moderate - severe (%)		18.0**	100	100	100	95	72

Numbers represent mean values (± SEM) and where given with the range of values in parentheses. The causes of death included bronchopneumonia (95%), cardiac arrest and carcinoma, renal failure and gastrointestinal bleed with no particular distribution pattern in any group. The post-mortem interval between death and tissue retrieval ranged 24–47 h for all the cases. There were no differences in the length of post-mortem delay between groups. Braak staging scores and Alzheimer’s Disease Neuropathologic changes [36] were different in mixed and AD cases compared to all other groups (‡*P* < 0.05). Mean vascular pathology scores (range) for PSND and PSD groups were 13.5 (13–14) and 13.3 (9–17) compared to 6.7 (0–10) for controls (†*P* < 0.05). These scores were derived as described previously, with white matter lesion (WML) pathology score assessed using the scale from Deramecourt et al. [9].. Mean WML Score was high in all post-stroke and dementia subjects compared to controls (†*P* < 0.01). WM/Vascular lesions had **P < 0.01 compared to all post-stroke and dementia subjects. Abbreviations: ABC: AD Neuropathology scoring system; AD: Alzheimer’s disease; APOE: apolipoprotein E; CAA: cerebral amyloid angiopathy; CAMCOG: Cambridge cognition examination; F: female; M: male; MMSE: Mini Mental state examination; N: number of subjects; na: not available; NPD: no pathological diagnosis; PSND: post-stroke non-demented; PSD: post-stroke dementia; VaD: vascular dementia; WM: white matter

### Brain tissues and neuropathological analyses

Neuropathological assessment was carried out as described previously [23]. We routinely used the following stains: Nissl and Luxol Fast Blue, haematoxylin and eosin (H&E), Bielschowsky’s and Gallyas. AD was clinically diagnosed on evidence of significant Alzheimer’s-type pathology incorporating Braak stages V–VI, moderate-severe CERAD [32] and high ABC scores, according to National Institute of Aging-Alzheimer’s Association guidelines [36], with the general absence of marked vascular pathology. The clinical diagnosis of vascular dementia (VaD) was made by the appearance of the following features: lacunae, multiple, cystic or border-zone infarcts, microinfarcts and small vessel disease, and could be pathologically confirmed as Braak stage ≤ IV [26, 28]. Cases were classified as Mixed AD and VaD when there was an abundance of both AD pathology [36] and significant vascular pathology present (Table 1). Vascular pathology including cerebral amyloid angiopathy scores and white matter lesion (WML) grading were assessed as described previously [9, 47]. Control subject tissues displayed occasional ageing-related pathology but were still classified as NPD or no pathological diagnosis (Table 1). Except for neuropathological examination (RNK, TMP), all subsequent morphological analyses were undertaken under operator-blinded conditions, with samples only identifiable as coded sequential numbers. Moreover, at least two positive and negative controls were included to monitor the quality of staining levels.

### Immunohistochemistry methods

Formalin-fixed paraffin-embedded coronal sections at 10 μm thickness cut from blocks of the whole hippocampus according to the Newcastle Brain Map [26, 42], which contained the CA1, CA2, CA3, CA4 and dentate gyrus (DG) hippocampal sub-regions were analysed. When sampling tissue, we ensured to select the hippocampal regions free of any apparent infarcts or gross lesions. Immunohistochemistry was performed to examine alterations across numerous microvascular structures effectively as was described before [10, 22]. The following antibodies were used to assess various cellular features and verify pericytes in this study: Drebrin A (DA at 1:400 dilution, Medical and Biological Laboratories Co., Ltd, Japan), F-actin binding protein localised in dendritic spines (post-synaptic), collagen IV (COL4 at dilution 1:1000, C1926, Sigma-Aldrich, Branchburg, NJ, USA), a marker of the basement membrane in the vessels, platelet-derived growth factor receptor-β (PDGFR-β at 1:200 dilution, clone 42G12, #AF385, R&D systems, Minneapolis, MN, USA), a marker for pericytes, α-smooth muscle actin (αSMA at dilution 1:1000, Clone 1A4, Dako, Cambridge, UK), a marker for mural cells, and glucose transporter-1 (GLUT-1 at 1:200, PA1-21041, Fisher Scientific, Waltham, MA, USA), a marker of endothelial cells. Vectastain ABC mouse kits (PK-6102, Vector Laboratories, Burlingame, CA, USA) and DAB were used to localise single immunohistochemical stains. Sections were then counter stained with haematoxylin to visualise landmarks across the tissue before mounting in DPX.

### Animals and surgical procedures

Adult male C57BL/6J mice (~ 25 g), purchased from Charles River, UK, were housed in groups on a 12 h day and 12 h night cycle (6am–6pm, day; 6pm–6am, night) and were given access to food and water *ad libitum*. A total of 74 mice were randomly selected for either bilateral common carotid artery stenosis (BCAS, *n* = 41) [21, 46] or sham (*n* = 33) surgery. Data analyses were performed under investigator blinded conditions by 2 or more observers. The surgical and animal housing procedures were pre-approved by the Home Office, UK based upon ASPA: The Animals (Scientific Procedures) Act 1986, UK and performed in accordance with the guidelines stipulated by the ethical committee of Newcastle University and also adhering to ARRIVE guidelines.

### Enriched environment (EE) in BCAS mice

One week after surgery, the BCAS and sham mice were randomly assigned to six subgroups, three different levels of EE per main group for 12 weeks: standard housing no EE, limited exposure to EE and full-time exposure to EE. Standard housing denotes normal housing conditions, which incorporated a paper house and shredded tissue. EE cages had extra gadgets in addition to the standard housing e.g. running wheels, hanging chains, igloos, and a paper tunnel. Limited exposure to EE was performed as described previously [29]. Briefly, for the first four weeks, mice were transferred to the EE cages for 3 h daily in the morning from 9am to noon. From 5th week to 12th week after BCAS surgery, mice experienced EE for 3 h, 3 days a week. Full-time EE group was exposed to EE every day for 24 h over the entire 12 weeks [21].

### COL4-specific immunocytochemistry to quantify pericytes

In accord with our work on the white matter and cortex [10, 11], we refined COL4 immunohistochemistry as a readily applied method to determine densities of capillary pericytes in disease and in experimental animal models. Tissue sections were immunostained with COL4 antibodies and then counterstained with haematoxylin. Nucleated pericytes were typically identified as nodules with “bumps” or “crescent” shaped cell bodies were counted manually along capillary profiles captured from more than 2500 images. The total number of pericyte cell bodies (> 2000) were then estimated for each case from 8 to 25 frames per case, with a mean number also calculated per case (Supplementary File 1). We encountered 10–17 nucleated pericyte cell bodies in each image, which ensured consistent counting methods. The pericyte soma was only included if it had the characteristic shape with a visible nucleus as identified by haematoxylin counterstain at 40X magnification. In preliminary experiments, we ensured bonafide pericytes were counted by demonstrating overlap between COL4 and PDGFR-β immunoreactivties, which clarified that COL4 + ve “bumps” were pericyte somata and negative staining for GLUT1 [10, 11]. We only quantified the pericyte cell density rather than pericyte cell coverage with processes because the aim was to assess potential alterations in pericyte nuclei and therefore pericyte cell status in dementia.

### Image acquisition and analysis

Regions of interest (ROI) within the tissue sections were captured as images on a Zeiss Axioplan 2.0 microscope and relevant image capture software (Infinity Capture V4.6.0, Lumenera Corporation), taking care to avoid larger arterioles > 50 μm external diameter. Immunohistochemical staining was then quantified by using Fiji Image software [45]. We assessed the hippocampal sub-region specific pericyte density per capillary length (cells/mm) as well as the percentage vascular area stained for COL4 (% COL4/Area mm^2^), which was measured for each case from at least 10 ROI images. Measures of atrophy for each respective hippocampal sub-region area was also quantified as a percentage of the total hippocampal area (% area/ total hippocampal area). Moreover, the quality of immunoreactvities between individual sections and cases was tested using the integrated optical density, which displayed no significant differences in values between disease and control subjects. We additionally found there was a lack of association between the groups for the immunohistochemical staining of COL4 and length of fixation, or post-mortem interval. The % COL4 area, capillary length and capillary diameter were all analysed manually using Fiji Image software [45]. All essential histopathological analyses were performed blind throughout to the operator.

### Statistical analyses

Data were analysed by using GraphPad Prism and SPSS (V19.0, IBM) statistical software and were confirmed as normally distributed using the Shapiro-Wilk test. Differences between means of groups were first tested using the appropriate one-way ANOVA followed by Tukey’s post-hoc test or Kruskal-Wallis H test. Linear correlations between the density of pericytes per capillary length (mm) and hippocampal sub-region atrophy (% sub-region volume/ total hippocampus volume) were performed using the Pearson’s correlation co-efficient, as was described previously [7]. Differences between groups were denoted as significant with a *P* value less than 0.05 and the data represents mean ± SEM.

## Results

### Hippocampal pathology and atrophy in dementia

**Fig. 1:**
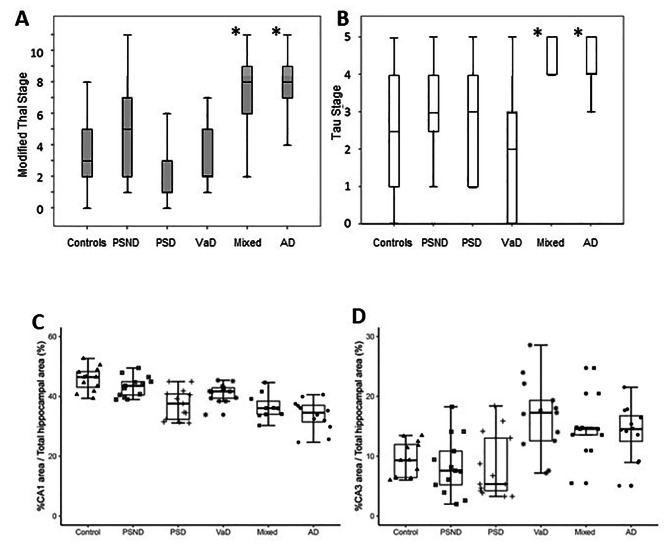
Hippocampal Pathology and Atrophy in Post-Stroke Dementia compared to other Dementias. **A-B**, Thal and Braak stage pathologies in different dementias compared to ageing controls and non-demented post-stroke survivors. There was significantly greater neurodegenerative pathology in Mixed (AD plus VaD) and AD cases *(P* < 0.01). **C-D**, Box plots showing hippocampal CA1 and CA3 areas relative to whole hippocampus in common dementias compared to ageing controls and non-demented post-stroke survivors. ANOVA and post-hoc tests showed that CA1 region atrophy across all dementias in PSD, VaD, Mixed and AD *(P* < 0.001). There was also a difference between PSD vs. PSND cases *(P* = 0.008). There were no significant differences in the CA3 region (D) or in CA4 or DG (not shown). Abbreviations: AD, Alzheimer’s disease, CA, cornus ammonis; DG, dentate gyrus, Mixed, mixed dementia VaD and AD; NPD, neuropathological diagnosis; PSD, post-stroke dementia; PSND, post-stroke no dementia; VaD, vascular dementia

The mean age and gender distribution of all the dementia subjects with relevant pathological findings are provided in Table 1. The available MMSE and CAMCOG scores indicated subjects had evidence of dementia at least 6 months prior to death. Figure 1 (A and B) shows high Thal scores and Braak stages in Mixed and AD groups compared to the vascular dementias, PSND and control groups. We found significant hippocampal formation atrophy [14] in the dementias and compared to controls. We found reductions in the % CA1 area indicative of atrophy in PSD *(P* < 0.001), VaD *(P* = 0.064), Mixed *(P* < 0.001) and AD *(P* < 0.001) subjects with the most severe changes occurring in PSD, Mixed and AD individuals. As expected, the PSND subjects also exhibited slight CA1 atrophy compared to ageing controls *(P* = 0.01). Thus, hippocampal atrophy was mainly driven by a 18–22% reduction in the CA1 region, which is the largest subfield of the hippocampal formation (Fig. 1C). In contrast, there was no evidence of significant change in size of the CA3 area (Fig. 1D) *(P* > 0.05) or the CA2, CA4 and DG regions (data not shown). Consistent with the neuronal atrophy and cellular changes in the CA1 region [17], we also found that the dendritic spine marker Drebrin A immunoreactivites were reduced across all the dementias compared to PSND and controls groups *(P* < 0.01) (Supplementary File 2).

### Hippocampal COL4-immunostained pericyte cell bodies

**Fig. 2:**
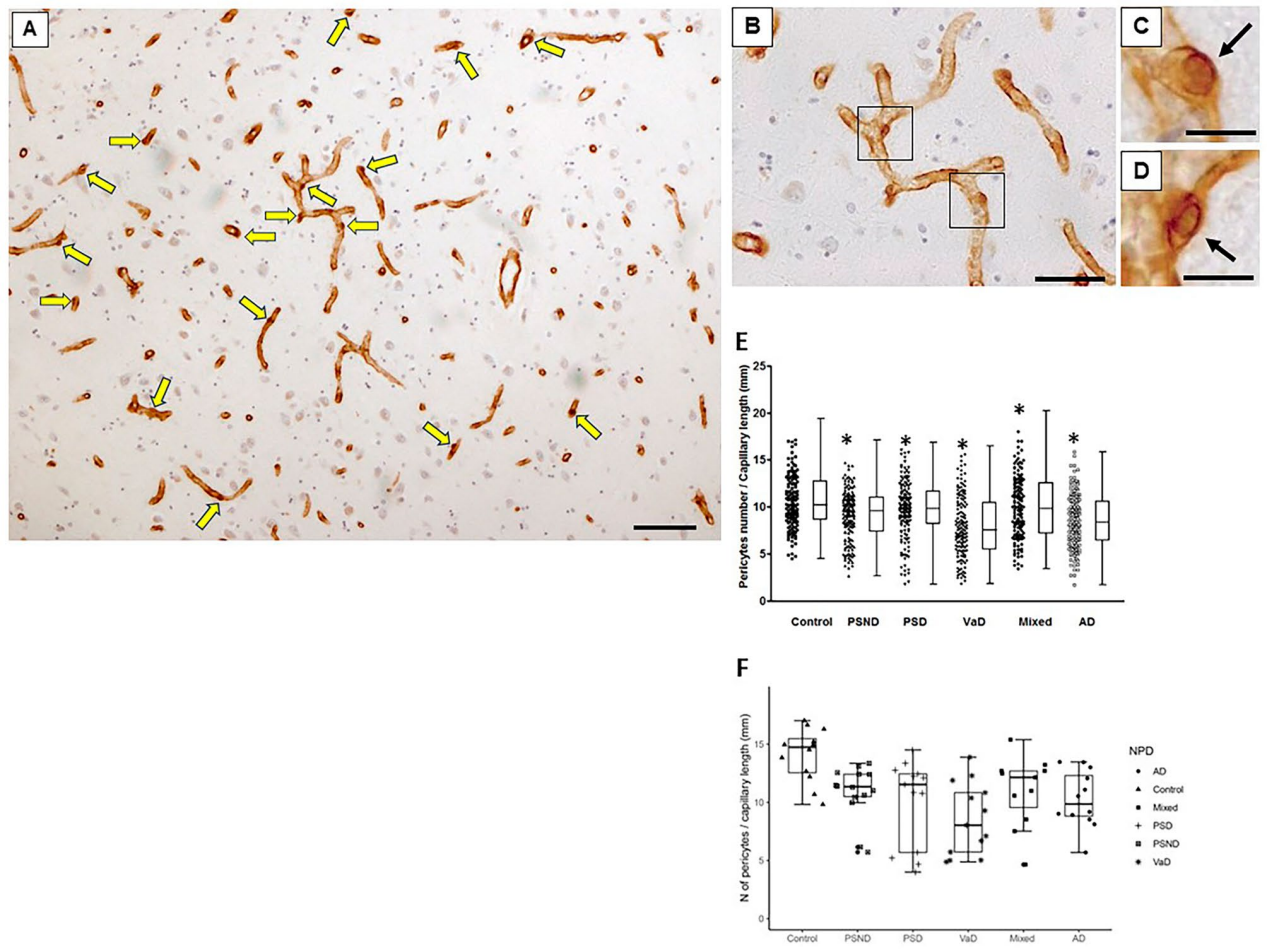
Quantification of hippocampal pericytes in dementias and ageing controls. **A-D**, Capillaries immunostained with COL4 showing pericytes identified by the morphology of ‘protrusion’ from the capillary walls surrounded by the COL4-positive membranes [10]. A, An original image at low power of the field containing 17 pericytes (yellow arrows). **B-D**, Images at higher power showing more detailed shape and size of capillary pericytes (black arrows). Only nucleated pericytes double positive for COL4 and haematoxylin were counted. **E-F**, Box plots showing number of pericytes per unit (mm) capillary length in the whole hippocampal formation (D) and CA1 region. The distribution of individual data points is shown beside each box plot. Pericyte numbers decreased in all groups compared to control subjects E, ***P < 0.001 Control vs. PSND and PSD; F, *P* < 0.01 Control vs. PSND and dementias. Scale bars; A-D = 50 μm.

As robustly demonstrated previously in the frontal cortex and white matter [10, 11], the appearance of “bumps” on a log or “crescent” shaped structures in hippocampal capillaries were COL4 immunopositive denoted as cell bodies of capillary pericytes (Fig. 2A-D). Their localisation to the abluminal surface of capillaries with envelopment of the COL4 immunostained basement membrane identified pericytes with diameter 7–9 μm. Pericyte cell bodies were higher compared to prior findings in the human neocortex [11] with range at 12–16 per mm length within the microvascular network.

Considering our prior analysis of cortical morphological changes within hippocampal cornu ammonis (CA) subfields, for this study we focused on the CA1 to CA4 plus the DG, which are associated with pyramidal neuron atrophy across different dementias [17, 18]. For the ageing controls, quantification of COL4 immunopositive pericyte cell bodies across the hippocampal sub-regions visualised with clear nuclei indicated that overall median densities were estimated to be 1280 cells per COL4 area mm^2^ and 12.8 cells per mm in capillary length. These estimates in the PSND group of stroke survivors without dementia were respectively 1120 and 11.2 per COL4 area mm^2^ and mm capillary length. This indicated a 12.5% reduction in the mean density per COL4 immunostained area and capillary length for PSND subjects relative to ageing controls. However, only the CA1 sub-region implicated the greatest reduction difference between PSND and controls for all hippocampal areas, as the reduction increases to ~ 20% suggesting an apparent predilection for CA1. There was no significant correlation between the loss of pericytes and any of the neurodegenerative pathology scores or the frequencies of apolipoprotein E (*APOE*) ε4 alleles (Table 1).

Pericyte somata densities within hippocampal regions across different dementias with a varying extent of vascular and neurodegenerative pathologies were found to be reduced (Fig. 2E). We found particularly for the CA1 area that irrespective of the metric used for estimation: mean density of pericytes per mm capillary length or per COL4 mm^2^ area, pericytes were significantly reduced for the dementia groups of PSD *(P* = 0.004), VaD *(P* < 0.001), Mixed *(P* = 0.028) and AD *(P* = 0.003) compared to age-matched elderly controls. Mean pericyte density in the CA1 per capillary length or capillary density for PSND subjects relative to controls was also decreased *(P* = 0.010) with apparent difference in pericyte density between PSND and PSD groups (Fig. 2F). We further noted lack of any notable changes for the % areas measured for COL4 immunostained capillary profiles in any of the dementia types apart from the mixed group slightly increasing in CA1 capillary density *(P* = 0.009), which was largely consistent with our prior findings in both the white matter and cortex [11, 22]. Additional analysis correlating the extent of CA1 atrophy against pericyte cell body numbers per capillary length (mm) did not reveal any strong associations across the PS survivors or dementia groups *(P* > 0.05) (Supplementary File 3).

**Fig. 3:**
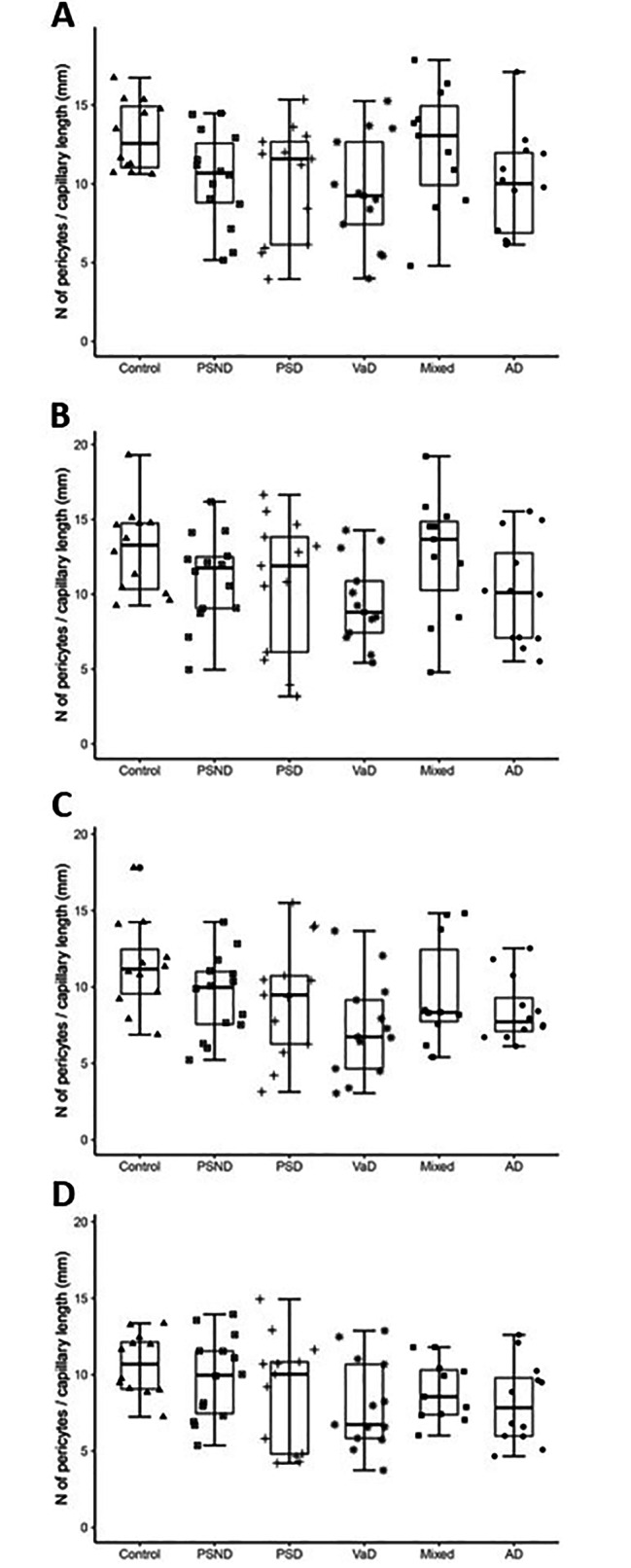
Quantification of hippocampal pericytes in the CA2, CA3, CA4 and DG regions across dementias. Box plots showing number of pericytes per unit (mm) capillary length in the CA2 (**A**), CA3 (**B**), CA4 (**C**) and DG (**D**). While there was variation in numbers of pericytes per region dependent on capillary densities there were no overall significant changes across the different dementia compared to controls *(P* > 0.05)

Pericyte densities per capillary length and capillary densities as well as region-specific density changes were also quantified for the CA2, CA3, CA4 and DG hippocampal subfields (Fig. 3A-D). However, no statistically significant changes were found in terms of contrasting disorder mean values (Fig. 3A-D). Pericyte numbers per capillary length did tend to decrease in PSND and dementia groups in the CA2, CA3 and CA4 except the DG. Correlation analysis for each specific hippocampal sub-region showed there was no apparent relationship between region area and pericyte densities *(P* > 0.05 for all regions). Thus, in terms of analyses for subfields of the hippocampal formation, only the CA1 region was found to be most vulnerable and susceptible to density changes within capillary pericyte somata.

### Hippocampal pericyte cell bodies in the BCAS model

**Fig. 4:**
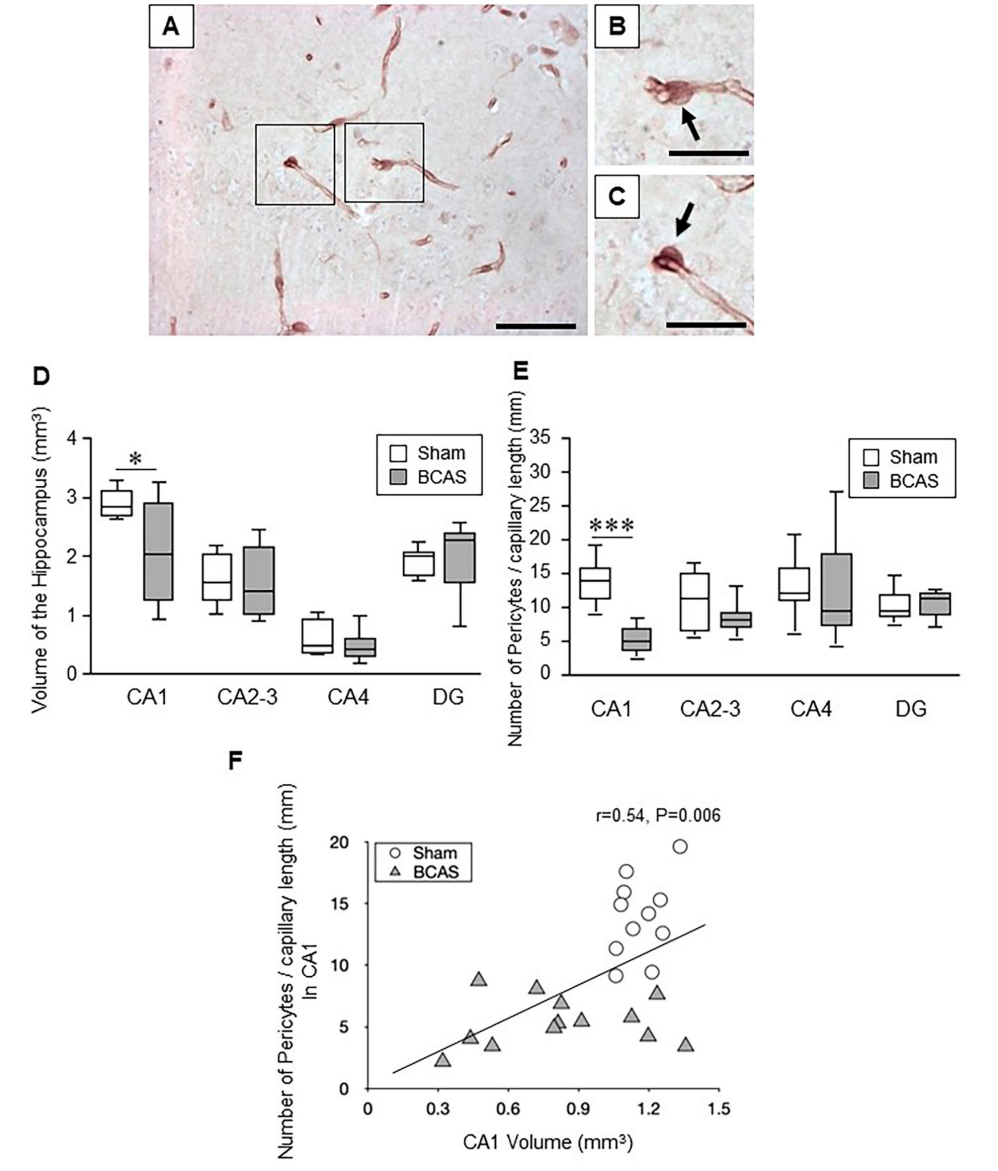
Assessment of capillary density, hippocampal volume and pericytes number in the hippocampus of BCAS mice. **A-C**, Representative images showing pericytes in the mouse hippocampus (arrows) subjected to bilateral common carotid artery stenosis (BCAS) using collagen IV (COL4) immunostaining. Scale bar = 50 μm (A); 25 μm (B-C). **D**, Box plots showing mean volume of the hippocampus in each subfield. BCAS caused CA1 atrophy (*P* = 0.018). **E**, Box plots showing number of pericytes per unit capillary length (/mm) in the hippocampus. Pericytes loss was evident in CA1 subfield of the hippocampus after BCAS (*P* = 0.000). **F**, Graph showing correlation between number of pericytes per unit capillary length (/mm) in CA1 and CA1 volume of the hippocampus after BCAS. Pericytes number per unit capillary length was positively correlated with CA1 volume (Pearson’s *r* = 0.54, *P* = 0.006)

**Fig. 5:**
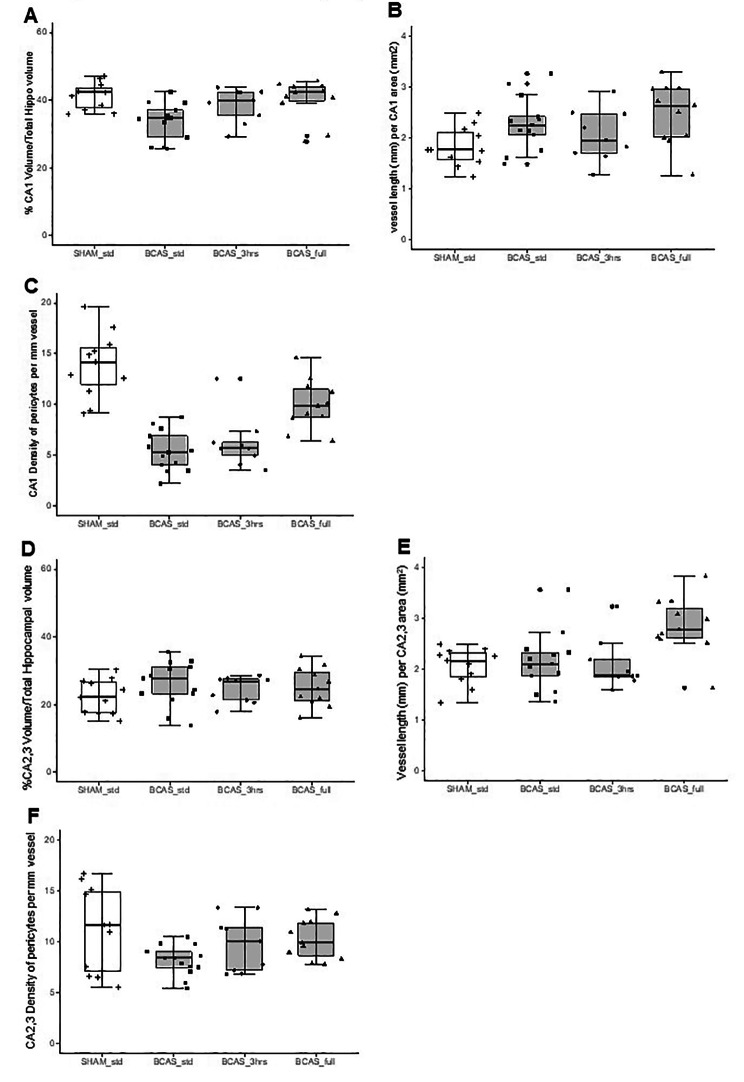
Quantification of pericytes per capillary length in the CA1 and CA2-3 regions in mice with BCAS. Box plots showing CA1 and CA2-3 volumes (**A**, **D**), vessel length (**B**, **E**) and the number of pericytes per unit (mm) capillary length (**C**, **F**). While there was variation in numbers of pericytes per region dependent on capillary densities there were no overall significant changes across the different dementia compared to controls (*P* > 0.05)

**Fig. 6:**
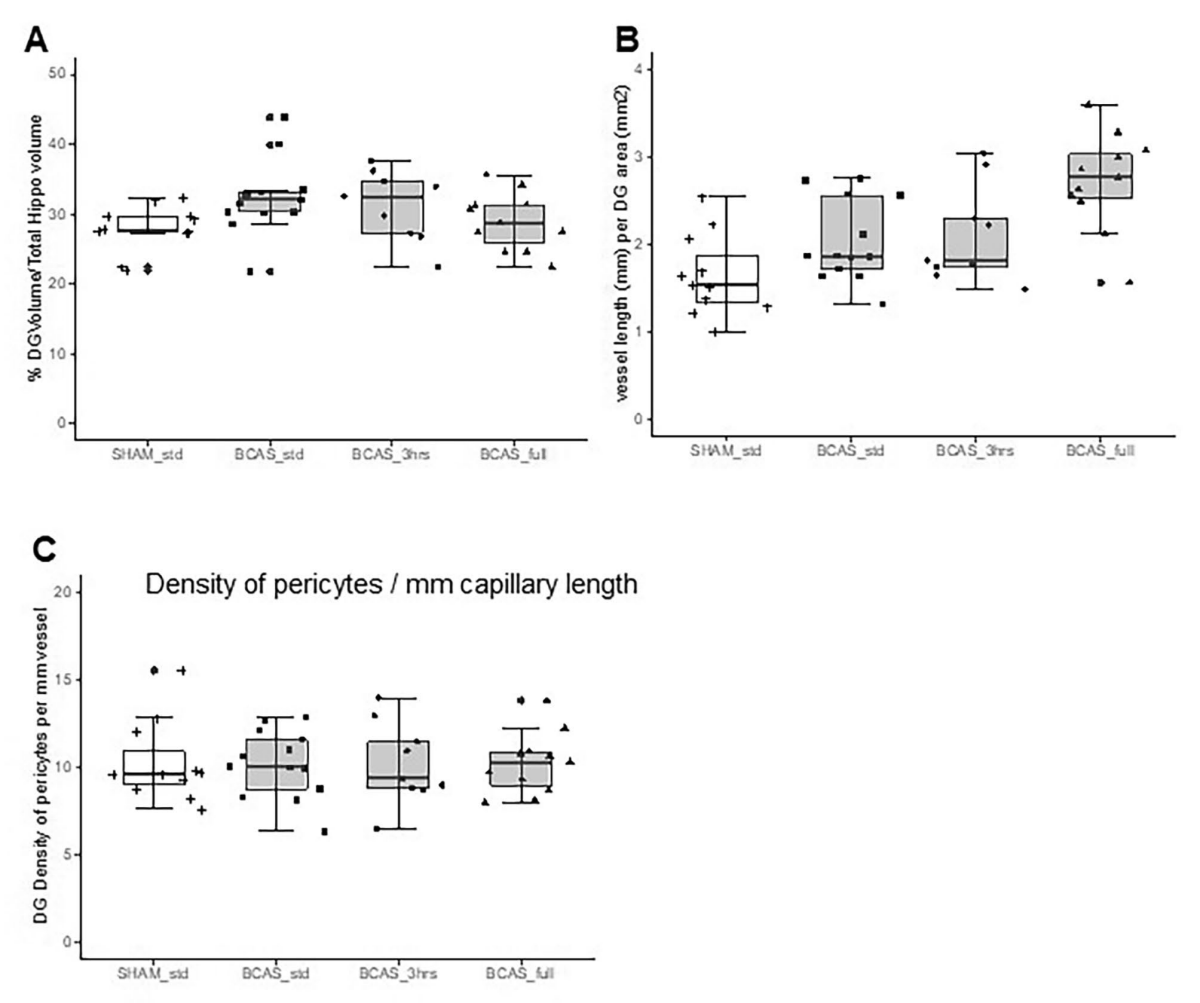
Quantification of pericytes per capillary length in the Dentate Gyrus (DG) in mice with BCAS. Box plots showing DG volume (**A**), vessel length (**B**) and number of pericytes per unit (mm) capillary length (**C**). While there was variation in numbers of pericytes per region dependent on capillary densities there were no overall significant changes across the different dementia compared to controls (*P* > 0.05)

As in the human hippocampus, we determined hippocampal densities of capillary pericytes in a similar manner in the BCAS mouse model, which mostly recapitulates VaD [23]. We have also previously shown that there is reduction in cerebral blood flow in the BCAS mouse model [23]. Capillary pericytes in the mice were 4–5 μm in diameter and located 13–15 per mm length (Fig. 4A-C). We found selective CA1 reduction in volume (atrophy) in the BCAS mice after chronic cerebral hypoperfusion *(P* = 0.008) (Fig. 4D). In the mouse, mean hippocampal capillary pericyte cell densities in sham animals were estimated to be 14 cells per mm vessel length in the order CA1 > CA2-3 > CA4 > DG (cf. Figures 4, 5 and 6). The density of CA1 pericytes measured per mm capillary length or per mm^2^ COL4 area was selectively reduced compared to the sham animals *(P* < 0.001) (Fig. 4E). Correlation analysis of all the data from the BCAS and sham groups showed a strong positive relationship between CA1 volume and numbers of pericyte per capillary mm length *(P* < 0.006) (Fig. 4F).

In further subfield analysis comparing standard BCAS group versus those that had 3 h EE and full-time EE, we first found that CA1 volumes were retained similar to sham controls (Fig. 5) in BCAS animals exposed to 3h or full-time EE (Fig. 5A). Moreover, both the relative CA1 capillary densities per mm length (or per CA1 area) were found to typically increase compared to the sham group by nearly 50% in the BCAS full treatment *(P* = 0.024) (Fig. 5B). Computing the capillary densities against pericytes numbers, we found remarkable recovery of pericytes in the EE animals, as a particularly strong effect in the full-time EE animals (Fig. 5C). CA1 pericyte densities were greater in the BCAS full EE treatment group compared to the BCAS std animal *(P* = 0.011).

Additional analyses revealed that the CA2/3 sub-region showed a reduction in pericyte density per capillary length (or per density) in the BCAS std group compared to sham animals *(P* = 0.041) but these were attenuated to sham control levels in the 3 h EE and full-time EE animals (Fig. 5F). We also found that capillary densities were increased in the full-time EE group *(P* = 0.027) but not significantly changed in the BCAS standard or 3 h EE group compared to sham animals (Fig. 5E). This latter finding of increased capillary density was replicated in the DG *(P* < 0.0001) (Fig. 6) in the absence of pericyte density changes (Fig. 6C). We also found no correlation between DG volumes and density of pericytes in individual animals in any of the groups including sham (Supplementary Fig. 4).

## Discussion

Given our prior studies on the white matter and cerebral cortex [10, 11], in this study we estimated numbers of nucleated pericytes in hippocampal sub-regions across ageing-associated vascular and neurodegenerative dementias, and normal controls. Notably, we found that pericyte cell body densities were approximately 3-fold greater within the hippocampal allocortex compared to the frontal cortex. Thus, median pericyte cell bodies per mm capillary length in normal anterior human hippocampus was 12.8 and those in the mouse was 14.0. To our knowledge, there are no previous estimations of such pericyte cell numbers for the hippocampus. More importantly in this study, we found that (1) there is loss of pericyte somata per capillary length in the whole hippocampal formation in both vascular and AD dementias, (2) the loss is remarkably sub-region specific, particularly the CA1 being most affected in dementia, (3) CA1 capillary pericytes are also lost in stroke survivors free of dementia (PSND group) or possibly before they develop dementia. This latter finding suggests remote stroke injury impacts on CA1 loss, likely due to early microvascular or perfusion changes modifying or remodelling the capillary network. Although the trends in lower number of pericytes in other hippocampal regions including the CA2 and CA3 may reflect intact nuclei it is possible the dynamic pericyte cell processes are undergoing retraction or modification [19]. However, overall these findings underscore profound vulnerability of the CA1, which appears to drive the shrinkage of the entire hippocampal structure. Our observations are also compatible with previous studies in which PDGFR-β immunoreactivity and soluble PDGFR-β were used to assess pericytes [35], particularly the earlier observations by Montagne et al. [34] showing that blood-brain barrier (BBB) breakdown in the CA1 region of the hippocampus worsened with mild cognitive impairment (MCI) and correlated with injury to BBB-associated pericyte. Interestingly, a recent experimental study suggests there is direct neuronal activity-driven signalling from insulin-like growth factor 2 expressing pericytes to neurons involved in learning and long-term memory [39]. It is plausible that that the CA1 loss of pericytes and of neurons we described previously [17] in similar cases could be related but this did not hold for the dentate gyrus [39].

Consistent with our previous observations on neuronal densities and arborisation [17, 18], we also found that irrespective of the type of pathology e.g. amyloid plaques, neurofibrillary tangles or microvascular changes within the hippocampal formation or remote to it there are similar losses in pericyte densities across dementias even though capillary densities remain unchanged or were increased. In view of the notable functions of the hippocampus in learning and memory, our findings overall suggest protection of the cerebral circulation and perfusion of the structure [25] by control of vascular risk factors is vital to prevent cognitive decline [8].

The BCAS mice experiments also remarkably showed that the hippocampal CA1 was most vulnerable with variable pericyte changes in other sub-fields. Chronic cerebral hypoperfusion caused by BCAS is non-invasive and therefore the changes in capillary structure and pericyte population appear entirely driven by likely haemodynamic alterations and perfusion deficits [24]. Similar findings compared to both those in human post-mortem tissue and BCAS mice were additionally prevalent with reduced hippocampal pericyte densities in a non-human primate model of chronic hypoperfusion induced by three vessel occlusion (*Ndung’u M, Hase Y, Kalaria RN, unpublished observations*). Thereby implicating that aberrant hippocampal pericyte somata changes in density are equally conserved across three distinct species and models of vascular-related or neuropathological dementia disorders.

Our most profound finding was the effect of EE on the hippocampal formation after BCAS. Outstandingly, full-time EE restored or attenuated effects on atrophy of the hippocampus as well as pericyte density per mm capillary length after chronic cerebral hypoperfusion. This finding emphasises how certain interventions are protective and beneficial for neurodegeneration and recurrent stroke injury that may act via neural and glial growth factors [21, 23, 30]. The question whether hippocampal function or pathophysiology can be modified in ageing and age-associated neurodegenerative and vascular dementias has been a topic of much research and discussion [16]. The role of physical activity or EE in improving hippocampal function and brain plasticity has been debated [12, 43] but, recent findings in various rodent models of degenerative disease are promising [31]. Our observations also support previous findings in patients with MCI that high intensity resistance exercise is capable of not only promoting better cognition, but also protecting dementia vulnerable hippocampal subfields from degeneration for at least 12 months post-intervention [5]. This could be explained by the hippocampal-specific cerebral blood flow effect of moderate post-exercise [38] although exercise-related vascular plasticity is highly variable among older adults indicating that other factors, such as the vascular network patterns in the medial temporal lobe may modify exercise-related benefits [49].

In individuals who develop cognitive impairment or dementia, the mechanism may be instigated by primary or focal endothelial damage which progresses [37]. It is plausible that a vicious cycle is set up whereby detachment of perivascular cells such as pericytes are exposed to blood-derived proteins which then creates a toxic environment on the abluminal side of the capillary network resulting in other cellular changes including clasmatodendrosis and microglial activation [21]. Previous in vivo two-photon imaging studies in the adult mouse cortex have shown that pericyte somata were immobile but the tips of their processes underwent extensions or retractions over days to cover bare regions of the capillary segment after selective ablation of single pericytes [4]. While removal of single pericyte soma in rodents does not affect focal BBB function, the absence of greater numbers of pericytes induces microvessel leakage and microvessel regression [35, 50] and almost the opposite scenario occurs when pericytes are implanted [48] in that cerebral blood flow is enhanced.

Our study has a few limitations. First, we did not assess pericyte numbers by 3D stereology but used a robust established method using wide screening with multiple sections in many cases. Our preliminary experiments demonstrated that it was unnecessary since we had previously shown that the qualitative changes described using 2D measurements are similar to those obtained with 3D stereology [6], which is immensely cumbersome for a large number of samples. While further labour-intensive work could reveal precise numbers and turnover of pericytes, we think our estimates of pericyte numbers are close to reality. The pertinent finding here is that despite an apparent lack of profound changes in capillary densities, hippocampal capillary pericyte somata were fewer in subjects who developed dementia. The availability of more specific markers of pericytes would also have been useful to corroborate our findings on the mechanics of pericyte cell impairment or turnover and determine if these are decreased intracellularly prior to complete degeneration in the persistently hypoperfusive state within the deeper structures of ageing-related dementias.

In summary, we found mostly CA1 region-specific loss of numbers of capillary pericytes in the hippocampus in ageing-associated dementia disorders. Pericyte cell loss is likely associated with age-related disintegration of the neurovascular unit of the hippocampus that impairs BBB function. These findings were replicated in the BCAS model of chronic cerebral hypoperfusion also associated with tissue volume loss. Remarkably, however, BCAS mice exposed to full-time EE exhibited restoration of not only CA1 structure volume and capillary density but also pericyte numbers. Our observations suggest that changes in tissue perfusion and local cellular needs modify capillary pericyte cell responses, which can be restored by appropriate interventions.

### Electronic supplementary material

Below is the link to the electronic supplementary material.

Supplementary Material 1

Supplementary Material 2

Supplementary Material 3

Supplementary Material 4

Supplementary Material 5

## Data Availability

The data that support the findings of this study are available on request from the corresponding author. The data are not publicly available due to privacy or ethical restrictions.
