# Supplementary figures and images for: Hippocampal capillary pericytes in post-stroke and vascular dementias and Alzheimer’s disease and experimental chronic cerebral hypoperfusion
Source: Acta Neuropathol Commun. 2024 Feb 15;12:29. doi: 10.1186/s40478-024-01737-8 (PMC10870440; doi:10.1186/s40478-024-01737-8)

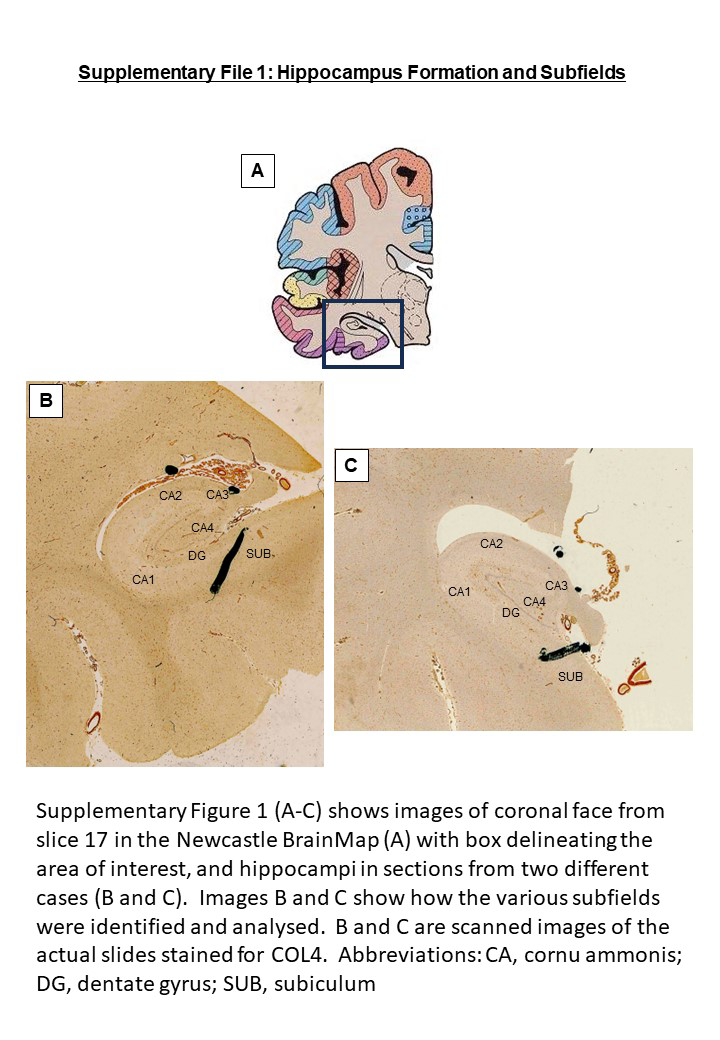

Supplement: Supplementary file 1 — Supplementary Material 1 [file 40478_2024_1737_MOESM1_ESM.png]

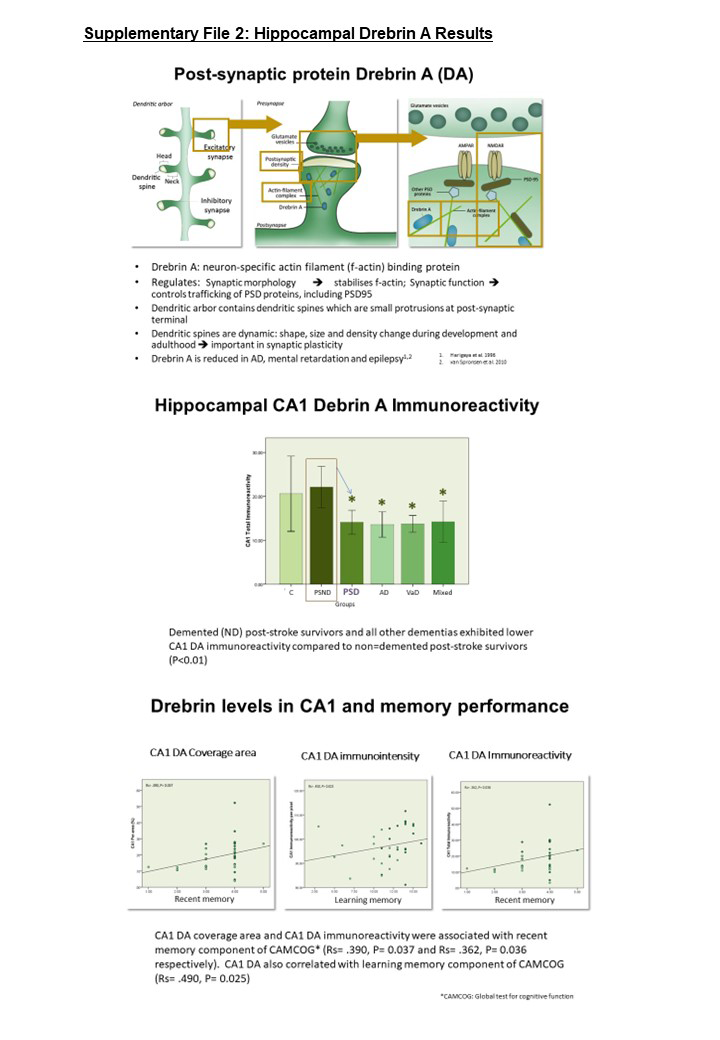

Supplement: Supplementary file 2 — Supplementary Material 2 [file 40478_2024_1737_MOESM2_ESM.png]

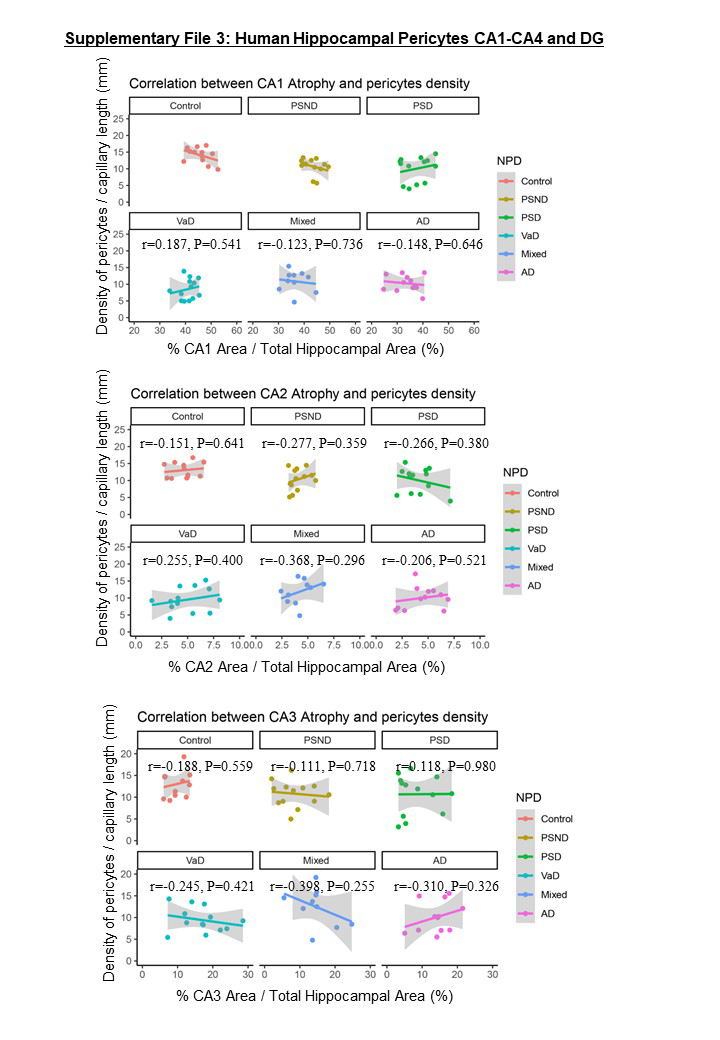

Supplement: Supplementary file 3 — Supplementary Material 3 [file 40478_2024_1737_MOESM3_ESM.png]

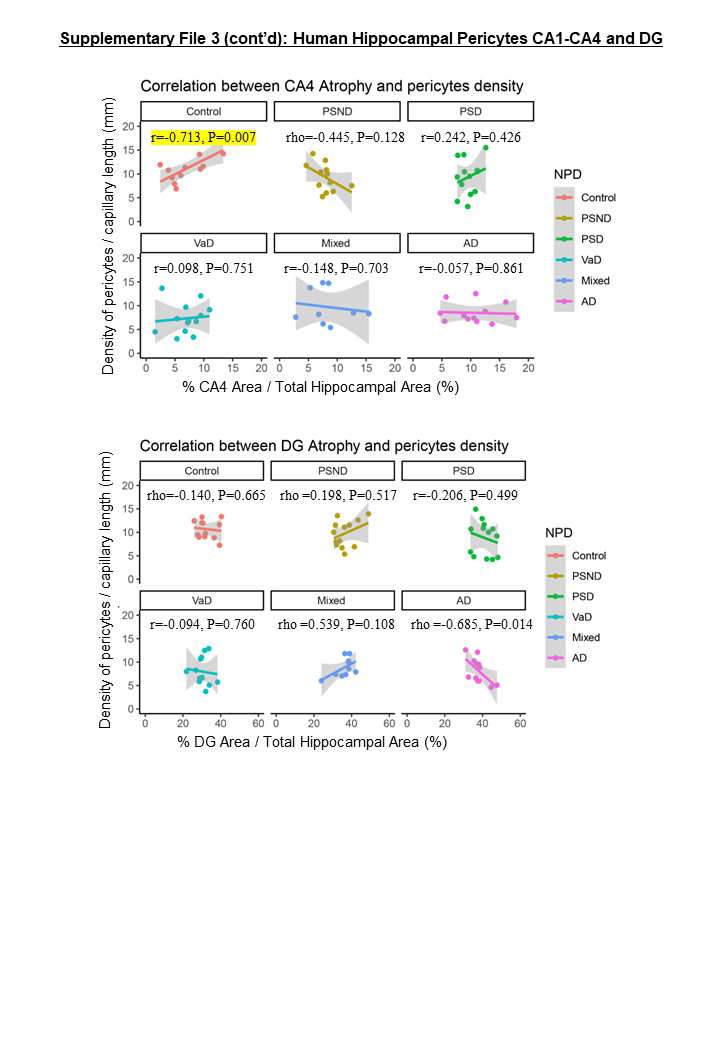

Supplement: Supplementary file 4 — Supplementary Material 4 [file 40478_2024_1737_MOESM4_ESM.png]

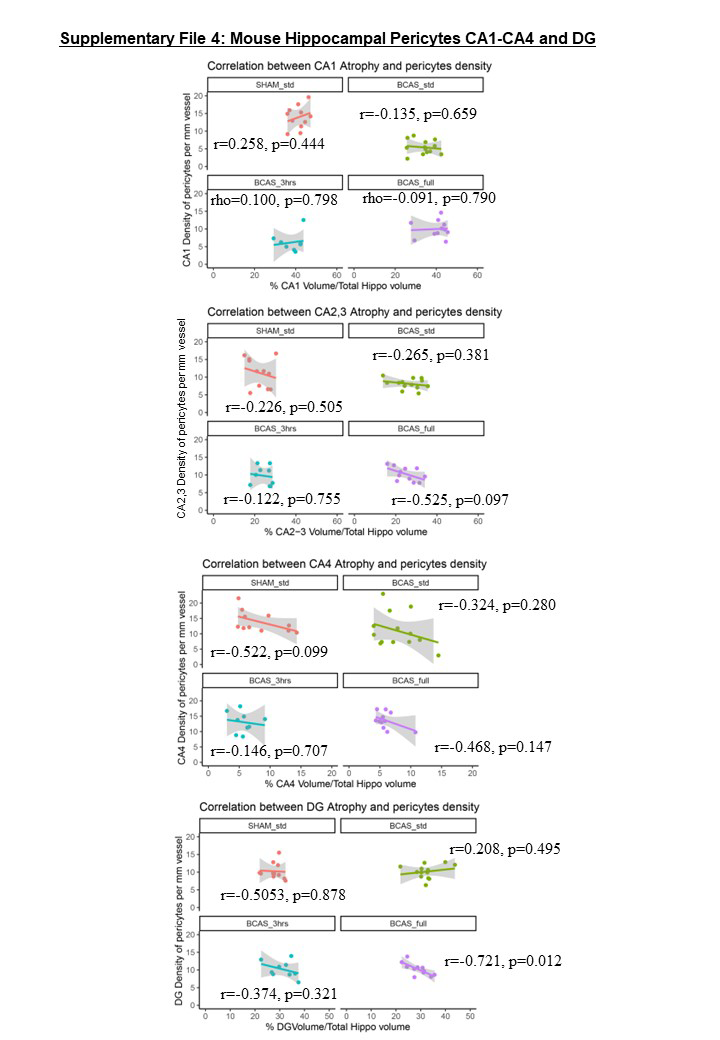

Supplement: Supplementary file 5 — Supplementary Material 5 [file 40478_2024_1737_MOESM5_ESM.png]
